# Signal Integration of IFN-I and IFN-II With TLR4 Involves Sequential Recruitment of STAT1-Complexes and NFκB to Enhance Pro-inflammatory Transcription

**DOI:** 10.3389/fimmu.2019.01253

**Published:** 2019-06-04

**Authors:** Anna Piaszyk-Borychowska, Lajos Széles, Attila Csermely, Hsin-Chien Chiang, Joanna Wesoły, Chien-Kuo Lee, Laszlo Nagy, Hans A. R. Bluyssen

**Affiliations:** ^1^Department of Human Molecular Genetics, Adam Mickiewicz University, Poznan, Poland; ^2^Department of Biochemistry and Molecular Biology, University of Debrecen, Debrecen, Hungary; ^3^Graduate Institute of Immunology, National Taiwan University College of Medicine, Taipei, Taiwan; ^4^Laboratory of High Throughput Technologies, Adam Mickiewicz University, Poznan, Poland; ^5^Departments of Medicine and Biological Chemistry, Johns Hopkins All Children's Hospital, Johns Hopkins University School of Medicine, St. Petersburg, FL, United States

**Keywords:** inflammation, interferons, TLR4, signal integration, atherosclerosis, JAK-STAT, STAT1 and NFκB

## Abstract

Atherosclerosis is a chronic inflammatory disease of the blood vessels, characterized by atherosclerotic lesion formation. Vascular Smooth Muscle Cells (VSMC), macrophages (MΦ), and dendritic cells (DC) play a crucial role in vascular inflammation and atherosclerosis. Interferon (IFN)α, IFNγ, and Toll-like receptor (TLR)4 activate pro-inflammatory gene expression and are pro-atherogenic. Gene expression regulation of many pro-inflammatory genes has shown to rely on Signal Integration (SI) between IFNs and TLR4 through combinatorial actions of the Signal Transducer and Activator of Transcription (STAT)1 complexes ISGF3 and γ-activated factor (GAF), and Nuclear Factor-κB (NFκB). Thus, IFN pre-treatment (“priming”) followed by LPS stimulation leads to enhanced transcriptional responses as compared to the individual stimuli. To characterize the mechanism of priming-induced IFNα + LPS- and IFNγ + LPS-dependent SI in vascular cells as compared to immune cells, we performed a comprehensive genome-wide analysis of mouse VSMC, MΦ, and DC in response to IFNα, IFNγ, and/or LPS. Thus, we identified IFNα + LPS or IFNγ + LPS induced genes commonly expressed in these cell types that bound STAT1 and p65 at comparable γ-activated sequence (GAS), Interferon-stimulated response element (ISRE), or NFκB sites in promoter proximal and distal regions. Comparison of the relatively high number of overlapping ISRE sites in these genes unraveled a novel role of ISGF3 and possibly STAT1/IRF9 in IFNγ responses. In addition, similar STAT1-p65 co-binding modes were detected for IFNα + LPS and IFNγ + LPS up-regulated genes, which involved recruitment of STAT1 complexes preceding p65 to closely located GAS/NFκB or ISRE/NFκB composite sites already upon IFNα or IFNγ treatment. This STAT1-p65 co-binding significantly increased after subsequent LPS exposure and correlated with histone acetylation, PolII recruitment, and amplified target gene transcription in a STAT1-p65 co-bound dependent manner. Thus, co-binding of STAT1-containing transcription factor complexes and NFκB, activated by IFN-I or IFN-II together with LPS, provides a platform for robust transcriptional activation of pro-inflammatory genes. Moreover, our data offer an explanation for the comparable effects of IFNα or IFNγ priming on TLR4-induced activation in vascular and immune cells, with important implications in atherosclerosis.

## Introduction

Atherosclerosis is a chronic inflammatory disease of the blood vessels, characterized by atherosclerotic lesion formation. Early onset of atherosclerosis is represented by recruitment of blood leukocytes to the injured vascular endothelium and altered contractility of Vascular Smooth Muscle Cells (VSMC) modulated by multiple inflammatory mediators (1). Accordingly, pro-inflammatory pathways activated by Toll-like receptors (TLRs), and Interferons (IFNs) have been identified as key components of atherogenesis (2–4). Type I (IFN-I; IFNα), and II (IFN-II; IFNγ) IFNs both induce IFN-stimulated gene (ISG) expression through Janus kinase (JAK)-dependent phosphorylation of Signal Transducer and Activator of Transcription (STAT)1. STAT1 homodimers, known as γ-activated factor (GAF), activate transcription in response to both IFN types by direct binding to IFN-II activation site γ-activated sequence (GAS)-containing genes. Association of Interferon Regulatory Factor (IRF)9 with STAT1–STAT2 heterodimers [known as Interferon-stimulated gene factor 3 (ISGF3)] in response to IFN-I, redirects these complexes to a distinct group of target genes harboring the Interferon-stimulated response element (ISRE) (5, 6). Limited evidence exists for a role of ISGF3 in IFN-II responses of ISRE-containing genes. Likewise, for a restricted number of ISGs, a non-canonical STAT1/IRF9 complex was shown to control IFNγ-responsiveness (7–9). The partially overlapping and differential activation of transcription factor complexes and regulation of target gene expression by IFN-I and IFN-II, may be a consequence of the biological similarities and differences of these two IFN types.

TLR4 ligation results in the prompt activation of multiple transcription factors, including members of the Nuclear Factor-κB (NFκB) and IRF families (10, 11). These factors rapidly induce the expression of hundreds of genes that amplify the initial inflammatory response, exert antimicrobial activities and initiate the development of acquired immunity. Several of the cytokines that are up-regulated in the initial wave of immediate early gene expression function in feed forward transcriptional loops—particularly important examples being IFN-I, which induce a secondary wave of STAT1- and STAT2-dependent gene expression, and Tumor necrosis factor (TNF) which sustains NFκB signaling.

Gene expression regulation of many pro-inflammatory genes has shown to rely on Signal Integration (SI) between IFNs and TLR4 through combinatorial actions of the STAT1-containing complexes ISGF3 and GAF with NFκB. For example, previous analyses of the murine *Nos2* promoter revealed an IFN response region (containing GAS and ISRE sites) and binding sites for NFκB (12). Indeed, sequential and cooperative contributions of NFκB preceding ISGF3 were shown to be involved in the transcriptional induction of the *Nos2* gene in macrophages (MΦ) infected with the intracellular bacterial pathogen *Listeria monocytogenes* (13). The *Nos2* gene reflects a larger group of genes, co-regulated by TLR4 and IFNs (14, 15). On the other hand, the profound effects of IFNγ pre-treatment (“priming”) on TLR4-induced MΦ activation have also long been recognized. In this respect, SI between IFNγ and lipopolysaccharide (LPS) relies on combinatorial actions of STAT1 with NFκB and IRFs on ISRE/NFκB or GAS/NFκB binding sites, which leads to enhanced transcriptional regulation of many pro-inflammatory genes. Together, this coordinates the antimicrobial and inflammatory responses in MΦ, but also in dendritic cells (DC) (16–19). Recently, we characterized the role of STAT1 in the transcriptional response pathways involved in the interaction between IFN-II and TLR4 signaling in endothelial cells (EC) and VSMC (20). Promoter analysis of the genes encoding multiple chemokines, adhesion molecules and antiviral and antibacterial response proteins, predicted that cooperation between NFκB and STAT1 is involved in the amplified transcriptional regulation of responses to IFN-II and LPS. The synergistic interactions between IFNγ and TLR4 also resulted in increased T-cell migration and impaired aortic contractility in a STAT1-dependent manner (20). Interestingly, expression of the *Nos2* gene in MΦ in response to IFNα/LPS behaved similar as after IFNγ/LPS (21), reflecting the existing overlap in activation mechanisms between the different types of IFN. However, the mechanistic role of SI between IFN-I and TLR4, in the context of “priming,” in vascular and immune cell has not been studied in much detail.

To characterize the mechanism of priming-induced IFNα + LPS- and IFNγ + LPS-dependent SI in vascular cells as compared to immune cells, we performed a comprehensive genome-wide analysis of VSMC, MΦ, and DC in response to IFNα, IFNγ, and/or LPS. Thus, through increased histone acetylation and RNA polymerase II (PolII) recruitment co-binding of transcription factor complexes activated by IFN-I or IFN-II together with LPS, including GAF, ISGF3, STAT1/IRF9, and p65-p50 heterodimers provide a platform for robust transcriptional activation of pro-inflammatory genes. Moreover, our data offer an explanation for the comparable effects of IFNα or IFNγ priming on TLR4-induced activation in vascular and immune cells, with important implications in atherosclerosis.

## Materials and Methods

### VSMC, MΦ, and DC Isolation

WT mice (strain background C57BL/6) were obtained from Charles River Laboratories. STAT1^−/−^ mice (strain background C57BL/6) (22) were kindly provided by Thomas Decker (Department of Microbiology, Immunobiology and Genetics, University of Vienna). Before any manipulations, animals were euthanized by cervical dislocation under isoflurane anesthesia. Primary VSMC were isolated from WT and STAT1^−/−^ mice aortas by enzymatic digestion (23). Briefly, aortas were dissected out and carefully cleaned from remnant fat and connecting tissue and cut into rings. Next, tissue was incubated with digestion mix consisting of DMEM [Thermo Fisher Scientific (TFS), 11960044] supplemented with 0.744 U/ml Elastase I (Sigma Aldrich, E1250), 1 mg/ml Collagenase II (Sigma Aldrich, 1148090) and 1 mg/ml soybean trypsin inhibitor (TFS, 17075029) for 1 h at 37°C. After digestion the cell suspension was passed through 100 μm cell strainer and left undisturbed for 1 week. Examination of marker gene (α-actin, smoothelin, calponin) expression by RT-PCR was used to assess VSMC cell phenotype. Freshly isolated femur and tibia form WT mice were cleaned from remnant muscle tissue by scrapping. Both ends of the bones were cut and bone-marrow was flushed and centrifuged for 5 min, 1,500 rpm. The cell pellet was incubated in ACK buffer (pH 7.2–7.4) in order to lyse red blood cells. Monocytes were purified through a Ficoll-Paque gradient (GE Healthcare, 17-1440). Afterwards primary MΦ were differentiated in DMEM medium [Thermo Fisher Scientific (TFS), 11960044] supplemented with 30% L929 conditioned medium (containing M-CSF), 15% FBS (Sigma-Aldrich, F7524) and 1:100 antibiotic/antimycotic solution (Sigma-Aldrich, A5955) for 5 days (24). Similarly, primary DC were differentiated from bone-marrow using a solution containing RPMI1640 medium (Sigma-Aldrich, R5886), 200 U/ml rmGM-CSF (PeproTech, 315–03), 10% FBS (Sigma-Aldrich, F7524), 1:100 antibiotic/antimycotic solution (Sigma-Aldrich, A5955), 2 mM L-glutamine (Sigma-Aldrich, 67513), and 50 μM β-ME (TFS, 31350-010) for 6 days according to a modified Lutz et al. protocol (25). Purity of MΦ and DC populations was assessed by flow cytometry, with F4/80 and CD11b, CD11c markers, respectively. Experimental procedures performed in this study, encompassing sacrificing mice for bone marrow or tissue isolation, did not require any medical ethical approval in accordance with the local legislation and institutional requirements.

### Cell Culture and Treatment

WT and STAT1^−/−^ VSMC were cultured in DMEM complete medium (TFS, 11960044) supplemented with 10% FBS (TFS, 10500-064), 1:100 L-glutamine (BioWest, X0550), and 1:100 antibiotic/antimycotic solution (Sigma-Aldrich, A5955). On the day before treatment, complete medium was exchanged onto 2% FBS containing starving medium. Differentiated MΦ and DC were immediately placed in serum free medium (TFS, 12065074) or 2% FBS (Sigma-Aldrich, F7524) containing RMPI1640 (Sigma-Aldrich, R5886) supplemented with 1:100 antibiotic/antimycotic solution (Sigma-Aldrich, A5955) and 50 μM β-ME (TFS, 31350-010), respectively, for 24 h. Afterwards, cells were treated with single stimulus as follows: 1,000 U/ml of IFNα (Merck Millipore, IF009) or 10 ng/ml of IFNγ (TFS, PMC4031) for 8 h; 10 ng/ml (MΦ and DC)/1 μg/ml (VSMC) of LPS (Sigma-Aldrich, L4391) for 4 h. To further study the effect of IFNs pre-treatment on LPS signaling the cells were first treated with IFNα or IFNγ, after 4 h LPS was added to the same cell culture plates for an additional 4 h, what resulted in a total of 8 h treatment with IFNs and 4 h treatment with LPS, at concentrations listed above. Described treatment strategy was applied in both RNA-seq and ChIP-seq experiments performed in this study.

### Gene Ontology (GO)

Protein ANalysis THrough Evolutionary Relationships (PANTHER) resource (26) was applied to identify statistically overrepresented GO terms for mapped lists of commonly up-regulated [Fold Change (FC) > 2] genes in VSMC, MΦ, and DC after combined treatment with IFNα + LPS (579 genes) and IFNγ + LPS (536 genes), using GO Biological Process Complete annotation data set. GO terms subjected for further comparison between the gene lists were selected as representative terms related to biological functions involved in immune, inflammatory, defense and stress response. Only GO terms with *p*-value of <0.05 were considered as significantly enriched.

### Promoter Analysis

Over-represented conserved Transcription Factor Binding Sites (TFBS) for STAT1 and NFκB were screened in the regulatory regions of commonly up-regulated (FC > 2) genes in VSMC, MΦ and DC after combined treatment with IFNα + LPS (579 genes) and IFNγ + LPS (536 genes) using pSCAN webserver (27). JASPAR Profiles for: GAS—MA0137.2, MA0137.3, ISRE—MA0652.1, MA0137.1, MA0.517.1, and NFκB—MA0105.1, MA0105.3. TFBS were analyzed in the region of −950/+50 bp to the nearest gene transcription start site. Applied threshold of matrix similarity score for potential GAS/ISRE and NFκB binding site was ≥0.85 and ≥0.90, respectively.

### Western Blot

Protein extracts from primary WT VSMC were prepared using Radio Immuno Precipitation Assay (RIPA) buffer (50 mM Tris-HCl, pH = 8.0 (Invitrogen, 15568025), 150 mM NaCl (Sigma-Aldrich, S9888), 1% Nonidet-40 (Bio-Shop, NON505), 0.5% sodium deoxycholate (Bio-Shop, DCA333), 0.1% SDS (Bio-Shop, SDS001), 1% protease inhibitor cocktail (Sigma-Aldrich, P8340), 1% EDTA (TFS, 15575-038), 0.1% PMSF (Sigma-Aldrich, 93482), and stored at −80°C. Protein concentrations were quantified using Bicinchoninic Acid (BCA) kit (Pierce, 23227). Sixty microgram of protein was heated in Bolt LDS buffer (Invitrogen, B0008) in 70°C for 10 min and loaded on Blot 4–12% Bis-Tris Plus Gels (Invitrogen, NW04120BOX), electrophoresed and transferred to PVDF membrane (GVS Nort America, 1231325). Western blot experiments were performed using SNAP ID Protein Detection System (Merck Millipore). Membranes were blocked either with 0.125% non-fat dry milk or with 1% BSA in TBS-Tween (TBS-T) and incubated with primary antibodies: tSTAT1 (CST, 14994, D1K9Y) 1:500, pSTAT1 (CST, 7649, D4A7) 1:500, tSTAT2 (CST, 72604, D9J7L) 1:400, pSTAT2 (Merck Millipore, 07-224) 1:500, IRF1 (CST, 8478, D5E4) 1:300, IRF9 (CST, 28845, D9I5H) 1:500, tp65 (CST, 6956, L8F6) 1:500, tubulin (Merck Millipore, 04-1117, EP1332Y) 1:2,000 and next with secondary HRP-conjugated antibodies: anti-rabbit (Sigma-Aldrich, A9169) 1:20,000, anti-mouse (Sigma-Aldrich, A9044) 1:20,000. Antibody-antigen complexes were visualized by enhanced chemiluminescence (ECL) using Luminata Forte HRP Substrate (Merck Millipore, WBLUF0500) and detected with G:Box System (Syngene). Image Studio Lite software (LI-COR Biosciences) was used for western blot quantification.

### Co-immunoprecipitation (Co-IP)

VSMC WT cells were lysed for 30 min in co-IP buffer [1% NP-40 (Bio-Shop, NON505), 150 mM NaCl (Sigma-Aldrich, S9888), 1 mM EDTA (TFS, 15575-038), 50 mM Tris HCl pH 7.5 (Invitrogen, 15567027) 10% Glycerol (Bio-Shop, GLY001)] supplemented with protease inhibitors. Cell lysates were immunoprecipitated with IRF1 (CST, 8478, D5E4) and IRF9 (CST, 28845, D9I5H) antibodies overnight at 4°C. Immunocomplexes were isolated with Dynabeads Protein A/G [TFS, 10008D(A), 10009D(G)] saturated with 1% BSA (Sigma-Aldrich, A3059), by gentle rocking for 3 h at. Beads were washed 3 times with ice-cold co-IP buffer and once with Tris-EDTA buffer. Next bound proteins were retrieved by boiling in Bolt LDS buffer (Invitrogen, B0008) for 10 min. Immunocomplexes were analyzed by Western blot (described in Materials and Methods section, Western blot) with tSTAT1 (CST, 14994, D1K9Y) 1:500 and tSTAT2 (CST, 72604, D9J7L) 1:400.

### RNA-seq Experimental Procedure

Total RNA from primary WT VSMC, WT MΦ, and WT DC treated as described above was isolated using GeneMATRIX Universal RNA Purification Kit (EURx, E3598). RNA-seq libraries were prepared from at least three biological replicates using a TruSeq RNA Library Preparation kit (Illumina, RS-122) according to the manufacturer's protocol. Libraries were quantified by Qubit fluorometer (TFS) and the quality was assessed with Agilent High Sensitivity DNA kit (Agilent Technologies, 5067-4626). Libraries were sequenced with Illumina HiScanSQ sequencer. To validate the quality of RNA-seq dataset, primary WT VSMC, WT MΦ, and WT DC were treated as described previously and 1 μg of RNA was used to synthetize complementary DNA with RevertAid Reverse Transcriptase (TFS, EP0441). *Cxcl9, Cxcl10, Ccl5, Nos2, Gbp6* transcripts were quantified using Maxima SYBR Green/ROX qPCR Master Mix (TFS, K0223) and CFX Connect Thermal Cycler System (Bio-Rad). Target gene levels were normalized to β-actin (ACTB) and quantified as described elsewhere (28) (described in Results section; data not shown).

### Chromatin Immunoprecipitation (ChIP)-seq Experimental Procedure

ChIP was carried out as previously described (29), with minor modifications. Briefly, primary WT and STAT1^−/−^ VSMC treated as described above were double-cross-linked with 0.5 M DSG (Sigma-Aldrich, 80424) for 45 min followed by 1% formaldehyde (TFS, 28906) for 10 min. Glycine (Sigma-Aldrich, G7126) was added for 10 min in 125 mM final concentration to stop cross-linking process. After fixation, nuclei were isolated by addition of ChIP Lysis Buffer (1% Triton X-100 (Bio-Shop, TRX777), 0.1% SDS (Bio-Shop, SDS001), 150 mM NaCl (Sigma-Aldrich, S9888), 1 mM EDTA (TFS, 15575-038), and 20 mM Tris, pH 8.0 (TFS, 15568-025). Chromatin was sonicated with Diagenode Bioruptor to generate fragments of 100–2,000 bp and immunoprecipitated with tSTAT1 (Santa Cruz, sc-346), pSTAT1 (CST, 7649, D4A7), tSTAT2 (CST, 72604, D9J7L), pSTAT2 (Merck Millipore, 07-224), IRF1 (CST, 8478, D5E4), IRF9 (CST, 28845, D9I5H), tp65 (CST, 6956, L8F6), RNA Polymerase II (Merck Millipore, 05-623, CTD4H8), Acetyl-Histone H3 (Lys27) (CST, 8173, D5E4), and Tri-Methyl-Histone H3 (Lys27) (CST, 9733, C36B11) antibodies. Following overnight incubation at 4°C, Dynabeads Protein A/G [TFS, 10008D(A), 10009D(G)] were added and incubated for 6 h at 4°C with rotation. Beads were washed at 4°C. DNA-protein complexes were eluted with Elution Buffer (1%SDS (Bio-Shop, SDS001), 0.1 M NaHCO3 (Sigma-Aldrich, S5761), and de-cross-linked with 0.2 M NaCl (Sigma-Aldrich, S9888) at 65°C. DNA was purified with MinElute PCR Purification kit (Qiagen, 28006) and quantified with Qubit fluorometer (TFS). ChIP-seq libraries were prepared from two biological replicates (for tSTAT1 and tp65 IPs) using TruSeq ChIP Library Preparation kit (Illumina, IP-202) according to the manufacturer's instructions. Libraries were quantified by Qubit fluorometer (TFS) and the quality was assessed with Agilent High Sensitivity DNA kit (Agilent Technologies, 5067-4626). Libraries were sequenced with Illumina HiSeq 2500 sequencer. Quality of ChIP-seq dataset was validated by ChIP-PCR experiments for selected STAT1 and p65 target genes (described in Results section; data not shown). All presented ChIP-PCR assays were performed using biological duplicates with primers listed in Table S2 (Supplementary Material). Statistical significance was estimated by two-way ANOVA and unpaired two-tailed student *T*-test.

### RNA-seq Data Analysis

RNA-seq raw sequence reads analysis was performed using Strand NGS software. After pre-alignment quality control (QC), alignment to the mouse mm10 (GRCm38) genome assembly was carried out using internal Strand NGS aligner which follows the Burrows-Wheeler Alignment (BWA) approach. All aligned reads were normalized using DESeq package. The data of the RNA-seq can be found at the NCBI GEO DataSets, with the accession number GSE120807. To determine differentially expressed genes (FC ≥ 2: up-regulated) gene lists were first filtered based on their normalized signal intensity values, with lower cut-off value>8. FC was calculated for these genes across different conditions and the resulting lists of up-regulated genes were used for the further downstream analysis. 18 lists (3 cell types × 6 conditions: control, IFNα, IFNγ, LPS, IFNα + LPS, IFNγ + LPS) of differentially expressed genes were compared and visualized using BioVenn diagram tool (30). Heatmaps presenting log2 transformed FC values for commonly up-regulated genes in VSMC, MΦ and DC after combined treatment with IFNα+LPS (579 genes) and IFNγ + LPS (536 genes) across control, IFNα, IFNγ, LPS, IFNα + LPS and IFNγ + LPS treatment conditions were generated using GraphPad Prism v.7 software.

### ChIP-seq Data Analysis

The primary analysis of ChIP-seq raw sequence reads was carried out using ChIP-seq analysis command line pipeline (31). Sequence reads were aligned to the mouse mm10 (GRCm38) genome assembly using the BWA tool (v0.7.10) (32), and bam files were created by SAMTools (v0.1.19) (32). Following converting mapped reads (bam files) by makeTagDirectory (HOMER v4.2 Hypergeometric Optimization of Motif EnRichment (33) to become accessible by the further HOMER tools, genome coverage (bedgraph) files were created by makeUCSCfile.pl (HOMER) (33) and converted to tiled data files (tdfs) by IGVtools (34). Peaks were predicted by MACS2 (v2.0.10) (*q*-value ≤ 0.01) (35), and artifacts were removed according to the blacklist of ENCODE (36). Intersections, subtractions, and merging of the predicted peaks (bed files) were made with BedTools (v2.23.0) (37). Tdf and bed files were visualized and genomic snapshots were taken with IGV2.3 (38). The closest gene for each peak was identified by annotatePeaks.pl (HOMER). The identification of DNA motifs was carried out in two steps. First, scanMotifGenomeWide.pl (HOMER) was used to identify all of the motifs genome-wide, specified by the publicly available motif files. Second, we determined the intersection between the identified motifs and peaks using intersectBed (bedtools). Sequencing data were submitted to NCBI GEO DataSets under accession number GSE120806.

### RD (Read Distribution) Plot Preparation

For clustering, occupancy values (expressed as Reads Per Kilobase Million, RPKM) were calculated for all STAT1 and p65 peaks. The peaks were clustered using k-means clustering (*n* = 10) based on the binding pattern of STAT1 and p65 in 6 samples (12 ChIP-seq data sets in total). Normalized tag counts for RD histograms were generated by HOMER and then visualized by Java TreeView.

### Peak Distribution Plot (Histogram) Preparation

Distances between summits of STAT1 and the closest p65 peaks summits were calculated using Phyton. Histograms were generated by annotatePeaks.pl from HOMER (with option-size 2,000 and -hist 25) and visualized by R using package ggplot2.

### Integrative RNA-seq and ChIP-seq Analysis

GAS, ISRE and NFκB-p65 consensus motifs from HOMER database (GAS—motif273, ISRE—motif140, NFκB—motif208; motif logos in Supplementary Material, Figure S1) were re-mapped to the called peak regions in STAT1 and p65 ChIP-seq experiments, after treatment with IFNα + LPS and IFNγ + LPS in VSMC. Next, list of 579 and 536 up-regulated commonly expressed genes in vascular and immune cells treated with IFNα + LPS and IFNγ + LPS, respectively, identified from RNA-seq experiment, were overlapped with the lists of re-mapped motifs regions. The lists of annotated genes containing re-mapped GAS, ISRE and NFκB motifs were initially filtered according to motif distance from the closest annotated gene TSS (–/+100 kb) and according to Motif Score Threshold (MST) (GAS—MST 6, ISRE—MST 6, NFκB—MST 7). Distribution of consensus GAS, ISRE, and NFκB binding sites occupied by STAT1 and p65 across the genome was classified into seven categories of genomic locations: promoter/TSS (−1 kb to +100 bp), introns, intergenic, exon, 5′ UTR, 3′ UTR, and TTS. Re-mapped motifs distribution was plotted by the percentage of total number of occupied GAS, ISRE and NFκB binding sites under treatment with IFNα + LPS or IFNγ + LPS.

## Results

### Commonly IFNα + LPS and IFNγ + LPS Regulated Genes Unravel Mechanistic and Functional Overlap of Priming-induced SI

**Figure 1:**
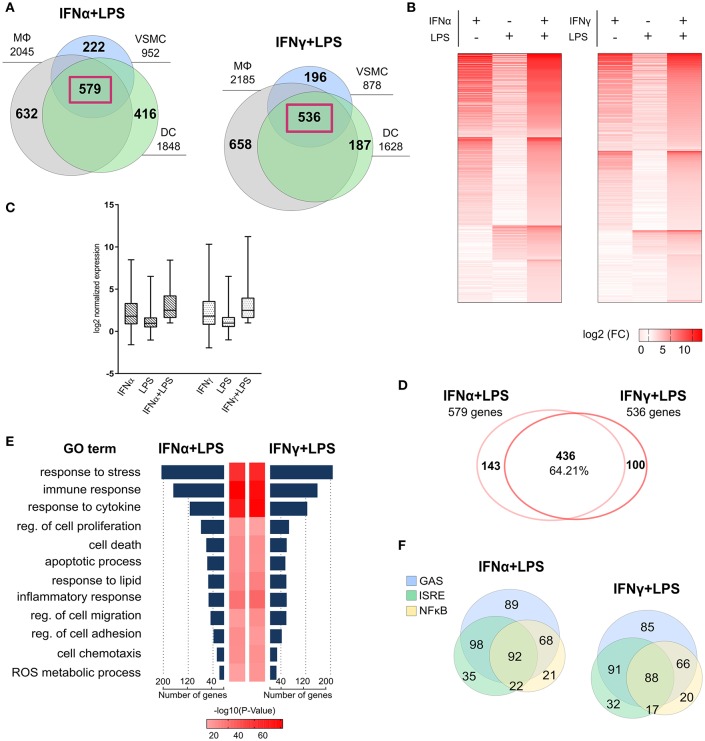
Mechanistic and functional characteristics of common gene expression between VSMC and MΦ, DC in response to IFNα + LPS and IFNγ + LPS. **(A)** Venn diagrams based on RNA-seq results showing intersection between lists of up-regulated (FC > 2) genes in VSMC, MΦ, and DC after combined stimulation with IFNα (8 h) + LPS (4 h) and IFNγ (8 h) + LPS (4 h). The gene lists for these Venn diagrams are shown in Table S1. Common 579 IFNα + LPS and 536 IFNγ + LPS-induced genes between three cell types were highlighted by violet frames. **(B)** Heatmap plots depicting expression pattern of commonly up-regulated, 579 IFNα (8 h) +LPS (4 h)- and 536 IFNγ (8 h) + LPS (4 h)-induced genes in VSMC, resulting from RNA-seq. Three main columns on each heatmap represent one particular treatment condition [IFNα (8 h), IFNγ (8h), LPS (4 h), IFNα (8 h) + LPS (4 h), or IFNγ (8 h) + LPS (4 h)]. Increasing brightness of red color indicates a higher gene expression level (gene expression is presented as log2 FC in comparison to control). **(C)** Box-plot representation of gene expression distribution for commonly up-regulated genes by IFNα (8 h) + LPS (4 h) and IFNγ (8 h) + LPS (4 h) resulting from RNA-seq (gene expression is presented as log2 FC in comparison to control) in VSMC. The line within each box represents the median and the lower and upper boundaries of each box indicate first and third quartiles, respectively. **(D)** Comparison of commonly up-regulated genes by IFNα (8h) + LPS (4 h) and IFNγ (8 h) + LPS (4 h) from RNA-seq, showing 64.21% overlap between the gene lists. **(E)** GO analysis of commonly up-regulated genes by IFNα (8 h) + LPS (4 h) and IFNγ (8 h) + LPS (4 h) (RNA-seq) revealed a strong enrichment for terms reflecting pro-inflammatory and pro-atherogenic biological functions. *P*-value < 0.05. **(F)** Venn diagram distribution of promoter located (−950/+50 bp) GAS, ISRE, and NFκB binding sites among commonly up-regulated genes by IFNα (8 h) + LPS (4 h) and IFNγ (8 h) + LPS(4 h) from RNA-seq experiment.

To characterize the mechanism of priming-induced IFNα + LPS- and IFNγ + LPS-dependent SI in vascular cells as compared to immune cells, we compared genome-wide transcriptional responses of VSMC, MΦ, and DC in response to IFNα (8 h), IFNγ (8 h), or LPS (4 h) alone, or after combined treatment (IFNα 8 h + LPS 4 h; IFNγ 8 h + LPS 4 h) using RNA-seq. Consequently, 579 genes were commonly up-regulated in VSMC, MΦ, and DC after combined treatment with IFNα + LPS (Figure 1A). Likewise, 536 genes were commonly expressed after combined treatment with IFNγ +L PS (Figure 1A). The complete lists of up-regulated genes in response to IFNα, IFNγ, or LPS alone, or after combined treatments in each cell type are shown in Table S1. To validate the quality of our RNA-seq dataset, the expression of a number of these genes, including *Cxcl9, Cxcl10, Ccl5, Nos2, Gbp6* was additionally confirmed by RT-PCR (data not shown).

Heatmaps presenting the expression pattern of the commonly 579 IFNα + LPS and 536 IFNγ + LPS regulated genes in VSMC, illustrate the potential effect of SI after combined treatment with IFNα + LPS or IFNγ + LPS as compared to the single stimuli (Figure 1B). Increasing brightness of red color in the heatmap reflects increasing gene expression levels, which in general are visibly higher after combined treatment with IFNα + LPS and IFNγ + LPS in comparison to single stimuli. After comparing the overall range and distribution of the common gene expression after single or combined stimulation (Figure 1C), in VSMC the effect of SI was clearly visible in the presented box plot. The median gene expression after combined treatment with IFNα + LPS and IFNγ + LPS was higher in comparison to single treatments with IFNs or LPS (Figure 1C). Tables 1A,B offer insight in the top-30 of these commonly up-regulated genes and illustrate the way they respond to IFNα + LPS and IFNγ + LPS in VSMC as compared to the single stimuli. The genes affected by SI (reflected by increased gene expression after combined treatment with IFNα + LPS or IFNγ + LPS vs. the sum of the single treatments; see Materials and Methods) are marked with an asterisk (Tables 1A,B). Strikingly, significant overlap could be observed between commonly up-regulated genes in response to IFNα + LPS and IFNγ + LPS. Indeed, the Venn diagram in Figure 1D shows 64.21% overlap between the 579 IFNα +LPS and 536 IFNγ + LPS commonly up-regulated genes (Figure 1D). Moreover, GO analysis of these 579 IFNα + LPS and 536 IFNγ + LPS commonly up-regulated genes revealed significant enrichment in overlapping terms connected to stress, immune and inflammatory response, response to cytokine, regulation of cell proliferation and migration, regulation of cell adhesion and chemotaxis, cell death and apoptotic process, response to lipid, and reactive oxygen species (ROS) metabolic process, all reflecting pro-inflammatory and pro-atherogenic biological functions. This also confirms the existence of functional overlap between vascular and immune cells, mediated by the interaction of both IFNs with LPS, which results in the execution of cell type-common biological responses (Figure 1E).

**Table 1A:** Representative top-30 genes commonly up-regulated (FC > 2) by IFNα + LPS in VSMC, MΦ, and DC, reflecting SI between IFNα and LPS in VSMC.

**No**.	**IFNα + LPS induced common genes**	**VSMC**			**Binding site**		
		**IFNα**	**LPS**	**IFNα + LPS**	**GAS**	**ISRE**	**NFκB**
1	F830016B08Rik^*^	196.8	6.4	345.9	•	•	–
2	Ifi44	356.3	4.4	343.7	–	•	–
3	Cxcl10^*^	75.9	4.6	312.1	•	•	•
4	BC023105^*^	187.1	19.9	289.2	–	–	–
5	Nos2^*^	4.9	90.9	287.1	•	•	•
6	Gm4955^*^	209.5	5.4	260.2	–	–	–
7	Gm15725	294.6	2.9	259.8	–	–	–
8	Iigp1^*^	179.7	9.2	242.3	•	•	–
9	Gm4951^*^	178.6	6.9	233.4	•	•	–
10	Gbp9^*^	78.8	22.9	215.9	–	•	•
11	Gbp11^*^	62.1	13.6	196.5	•	•	–
12	Apod	213.7	2.2	194.9	–	•	•
13	Gm4841^*^	99.8	9.0	181.4	•	•	–
14	Gbp4^*^	36.4	16.4	162.0	•	•	•
15	Gm14446	164.5	1.8	157.8	–	–	–
16	Mx1^*^	128.8	3.3	155.9	–	•	•
17	Ifit1^*^	113.3	11.1	153.6	–	•	–
18	Gm12250^*^	94.8	3.6	142.3	•	•	–
19	Gm4902^*^	126.5	3.8	139.3	–	–	–
20	Tnfsf10^*^	35.8	3.2	128.9	•	•	•
21	Usp18^*^	96.3	8.9	127.5	–	•	•
22	Gbp1^*^	91.7	17.5	125.5	–	•	–
23	Gbp6^*^	36.9	23.5	117.2	•	•	•
24	Gbp10^*^	35.6	21.1	115.2	–	•	•
25	Ch25h^*^	3.5	19.6	112.6	•	•	•
26	Tgtp2	114.2	2.6	110.7	•	•	–
27	Gm6904^*^	94.9	2.6	107.8	–	•	–
28	Zbp1^*^	95.5	5.8	106.3	•	•	•
29	Saa3^*^	5.1	66.6	105.7	•	–	•
30	Phf11^*^	100.2	3.2	105.7	–	–	–

Gene expression levels were presented as FC relative to control in VSMCs. Signal Integrated genes (FC IFNα + LPS > FC IFNα + FC LPS) were marked by an asterisk (

* *). Overlapping genes between IFNα + LPS- and IFNγ + LPS-induced commonly up-regulated genes (Table 1B) were color-coded by blue. Presence of GAS, ISRE, or NFκB binding sites in the promoters of listed genes was indicated by a dot (•)*.

**Table 1B:** Representative top-30 genes commonly up-regulated (FC > 2) by IFNγ + LPS in VSMC, MΦ, and DC, reflecting SI between IFNγ and LPS in VSMC.

**No**.	**IFNγ + LPS induced common genes**	**VSMC**			**Binding site**		
		**IFNγ**	**LPS**	**IFNγ + LPS**	**GAS**	**ISRE**	**NFκB**
1	Cxcl9^*^	82.2	4.0	2380.5	•	–	•
2	F830016B08Rik^*^	1272.1	6.4	2306.6	•	•	•
3	Gm4841^*^	1087.3	9.0	1650.3	•	•	•
4	Nos2^*^	1.8	90.9	933.3	•	•	•
5	BC023105^*^	600.8	19.9	909.4	–	–	–
6	Gbp4^*^	304.3	16.4	795.8	•	•	•
7	Iigp1^*^	687.7	9.2	779.3	•	•	–
8	Ubd^*^	95.6	5.8	655.1	•	•	•
9	Gbp10^*^	315.9	21.1	588.2	•	•	•
10	Gbp9^*^	304.8	22.9	586.1	–	•	•
11	Gbp6^*^	266.1	23.5	555.3	•	•	•
12	Serpina3f^*^	200.1	13.0	529.6	•	•	•
13	Gbp11^*^	302.3	13.6	482.7	•	•	–
14	Gm12250	502.9	3.6	477.8	•	•	–
15	Gbp8^*^	215.5	12.9	405.2	•	•	•
16	Ciita	704.4	2.0	376.5	•	•	•
17	Cxcl10^*^	49.8	4.6	364.9	•	•	•
18	Gbp1^*^	295.0	17.5	364.8	–	•	–
19	Gja4^*^	82.3	1.6	329.9	•	•	•
20	Gm4951^*^	300.1	6.9	327.2	•	–	–
21	Batf2^*^	191.4	3.1	298.4	•	•	–
22	Lcn2^*^	3.8	36.5	289.2	–	–	•
23	Gbp2^*^	219.7	13.4	284.4	–	•	•
24	Igtp	328.8	5.3	274.1	•	•	–
25	Tgtp2	261.9	2.6	262.5	•	•	–
26	Gm5970^*^	183.1	2.3	236.1	–	–	–
27	Ccl8^*^	115.8	18.6	231.4	•	•	•
28	Tgtp1^*^	216.7	2.4	222.4	•	•	•
29	Gbp5^*^	67.7	9.2	211.5	•	•	•
30	Saa3^*^	4.2	66.6	196.1	•	–	•

Gene expression levels were presented as FC relative to control in VSMCs. Signal Integrated genes (FC IFNγ + LPS > FC IFNγ + FC LPS) were marked by an asterisk (

* *). Overlapping genes between IFNγ + LPS- and IFNα + LPS-induced commonly up-regulated genes (Table 1A) were color-coded by blue. Presence of GAS, ISRE, or NFκB binding sites in the promoters of listed genes was indicated by a dot (•)*.

On the same lists of IFNα + LPS and IFNγ + LPS commonly up-regulated genes we also performed *in silico* promoter analysis, for the presence of ISRE, STAT, or NFκB binding sites in the proximal promoter (−950 to +100 bp). The predicted representation of individual or combined GAS, ISRE, or NFκB binding sites is depicted in Figure 1F. Most of the genes contained either single GAS sites (89 IFNα + LPS genes and 85 IFNγ + LPS genes) or rather combinations of potential GAS-ISRE (98 IFNα + LPS genes and 91 IFNγ + LPS genes), GAS-NFκB (68 IFNα + LPS genes and 66 IFNγ + LPS genes), ISRE-NFκB (22 IFNα + LPS genes and 17 IFNγ+LPS genes), or GAS-ISRE-NFκB (92 IFNα + LPS genes and 88 IFNγ + LPS genes) binding sites. Together this suggested that a common SI mechanism is involved in the interaction between IFNα and LPS or IFNγ and LPS in VSMC, in analogy to MΦ and DC.

### Genome-wide Binding of STAT1 and p65 to IFNα + LPS and IFNγ + LPS Regulated Genes Is Mediated Through Comparable Single and Co-binding Modes

To obtain further insight in the mechanism of priming-induced SI between IFNs and LPS in VSMC, we characterized the genome-wide binding of STAT1 and NFκB (p65) to the regulatory regions of IFNα + LPS and IFNγ + LPS commonly up-regulated genes. Thus, we performed ChIP-seq on chromatin from VSMC exposed to IFNα (8 h), IFNγ (8 h), or LPS (4 h) alone, or combined treatment (IFNα 8 h + LPS 4 h; IFNγ 8 h + LPS 4 h).

**Figure 2:**
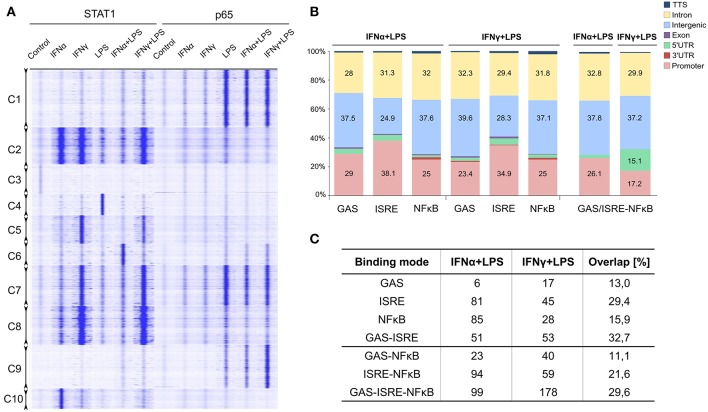
Genome-wide role of STAT1 and p65 in transcriptional regulation of commonly up-regulated IFNα + LPS- and IFNγ + LPS-induced genes. **(A)** RD heatmaps for ChIP-seq peaks clustered (*k*-means clustering) based on STAT1 and p65 binding pattern across control, IFNα (8 h), IFNγ (8 h), LPS (4 h), IFNα (8 h) + LPS (4 h), IFNγ (8 h) + LPS (4 h) treatment conditions in VSMC. Identified clusters are marked as Cluster (C) 1–10. **(B)** Global distribution of STAT1 (GAS and ISRE) and p65 (NFκB) occupied binding sites (from ChIP-seq) in 7 genomic locations (color-coded with the mapping provided in the legend): promoter/TSS (−1 kb to +100 bp), introns, intergenic, exon, 5′ UTR, 3′ UTR, and TTS. Re-mapped motifs distribution was plotted by the percentage of total number of occupied GAS, ISRE, and NFκB binding sites present in the regulatory regions of commonly up-regulated IFNα (8 h) + LPS (4 h)- and IFNγ (8 h) + LPS (4 h)-induced genes. **(C)** Representation of STAT1 and p65 occupied binding sites identified by ChIP-seq, representing “single” modes (STAT1 binding to GAS and/or ISRE; p65 binding to NFκB) or “co-binding” modes (STAT1 binding to GAS and/or ISRE together with p65 to NFκB). Table depict number of the genes within each STAT1/p65 binding mode among IFNα (8 h) + LPS (4 h)- and IFNγ (8 h) + LPS (4 h)-induced genes together with percentage overlap between the two treatment conditions.

Clustering analysis of the genomic regions occupied by STAT1 and/or p65 in response to single or combined treatments (Figure 2A) are visualized as tag counts (blue signals) in the RD plot. This analysis revealed that a subset of STAT1 and p65 binding regions (i.e., Cluster 7) were clearly co-occupied by these transcription factors when combined treatments (IFNα + LPS or IFNγ + LPS) were used, reflected by increased blue color intensity on the graph (Figure 2A). However, other genomic regions correlated with binding of STAT1 or p65 alone [i.e., Cluster C1, C9 (p65 only), Cluster C2 and C8 (STAT1 only)]. Subsequently, using HOMER software, GAS, ISRE, and NFκB consensus motifs (Figures S1A–C) were re-mapped to STAT1 and p65 binding regions and compared to the lists of 579 IFNα + LPS and 536 IFNγ + LPS commonly up-regulated genes (Figure 2B). Genomic binding analysis indicated that the STAT1 (GAS or ISRE) and p65 (NFκB) binding sites were primarily located in distant intergenic regions and intronic regions, while to a lesser extent in promoters, of IFNα + LPS- and IFNγ + LPS-responsive genes (Figure 2B). A similar distribution could be observed for the location of STAT1-NFκB co-binding sites (Figure 2B), which is in agreement with the above presented promoter analysis (Figure 1F), and predict the presence of multiple STAT1 and NFκB binding sites in the promoters of the IFNα + LPS and IFNγ + LPS commonly up-regulated genes.

By next comparing genome-wide binding results for STAT1 and p65 after VSMC stimulation with IFNα + LPS and IFNγ + LPS, we could identify different groups of genes, where STAT1 bound to consensus ISRE and/or GAS sites and p65 to NFκB sites. These binding sites were present in gene regulatory regions and existed in different combinations. As such we could distinguish genes which contained solitary ISRE, GAS, or NFκB sites, but also GAS-ISRE, ISRE-NFκB, GAS-NFκB, or GAS-ISRE-NFκB sites. Based on these gene groups we further defined STAT1 and p65 binding modes, including “single” (STAT1 binding to GAS and/or ISRE; p65 to NFκB) or “co-binding” (STAT1 binding to GAS and/or ISRE + p65 to NFκB) (Figure 2C). Among IFNα + LPS-induced genes, 6 GAS-only, 81 ISRE-only, 85 NFκB-only, and 51 GAS-ISRE containing genes, were identified. In case of IFNγ + LPS stimulation, we could distinguish 17 GAS-only, 45 ISRE-only, 28 NFκB-only, and 53 GAS-ISRE containing genes. Together they reflect the “single” binding mode. In addition, IFNα + LPS- and IFNγ + LPS- induced genes also included STAT1-p65 “co-binding” genes, which could be divided in GAS-NFκB: 23 and 40 genes, ISRE-NFκB: 94 and 59 genes and GAS-ISRE-NFκB genes: 99 and 178 genes, respectively.

Comparison of the different binding modes between IFNα + LPS- and IFNγ + LPS-induced conditions, identified a substantial overlap for NFκB-only (15.9%), GAS-only (13%), and ISRE-only (29.4%) containing genes from the “single” mode (Figure 2C). As reported previously, both IFN-I and IFN-II direct GAF complexes to GAS motifs, what is reflected by 13% overlap between the two conditions within GAS-only mode in our study. Yet 29.4% overlap found between IFNα- and IFNγ-activated genes within ISRE-only mode was very surprising, since limited evidence exists for a role of ISGF3 in IFN-II-driven gene expression. Likewise, this overlap could be observed for GAS-ISRE (32.7%), GAS-NFκB (11.1%), ISRE-NFκB (21.6%), and GAS-ISRE-NFκB genes (29.6%) from the “co-binding” mode (Figure 2C).

Collectively, this suggests that a common genome-wide SI mechanism exists, which involves combinatorial actions of ISGF3 or GAF with NFκB on ISRE/NFκB or GAS/NFκB binding sites, in the interaction of IFNα and LPS or IFNγ and LPS in VSMC.

### STAT1 as Part of ISGF3 Regulates Transcription of ISRE-containing Genes in Response to IFN-I and IFN-II

**Figure 3:**
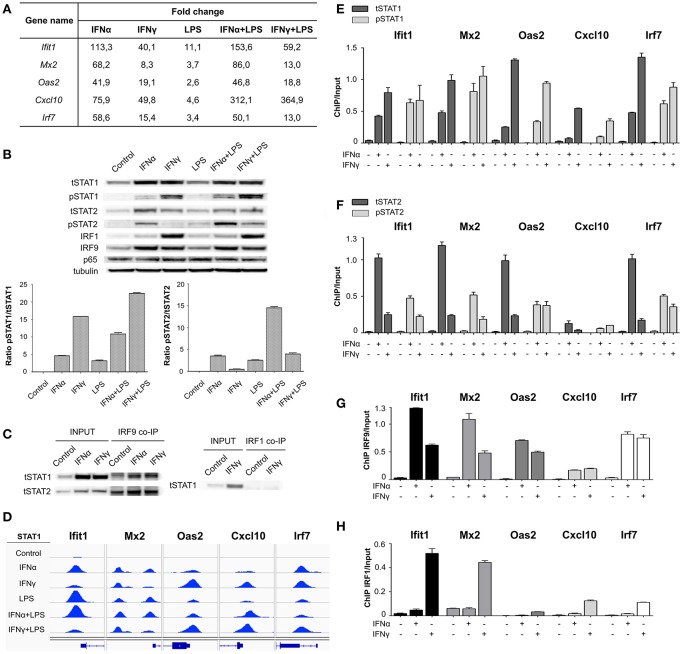
STAT1, STAT2, IRF9, and IRF1 in transcriptional regulation of ISRE-containing genes under stimulation with IFN-I and IFN-II. **(A)** Gene expression values (FC in comparison to control) for *Ifit1, Mx2, Oas2, Cxcl10*, and *Irf7* genes, resulting from RNA-seq: VSMC untreated or treated with IFNα (8 h), IFNγ (8 h), LPS (4 h), IFNα (8 h) + LPS (4 h), IFNγ (8 h) + LPS (4 h). **(B)** Western blot. Protein extracts were isolated from VSMC untreated or treated with IFNα (8 h), IFNγ (8 h), LPS (4 h), IFNα (8 h) + LPS (4 h), IFNγ (8 h) + LPS (4 h). Levels of tSTAT1, pSTAT1, tSTAT2, pSTAT2, IRF1, IRF9, p65, and tubulin were assessed by Western blot. *n* = 3, one representative blot is presented; Western blot quantification. Bars represent mean quantification of pSTAT1/tSTAT1 and pSTAT2/tSTAT2 ratio (normalized to tubulin). Mean ± s.e.m., *n* = 3; **(C)** Co-IP. Protein extracts were isolated from VSMC untreated or treated with IFNα (8 h) and IFNγ (8 h), immunoprecipitated with IRF9 or IRF1 antibodies and analyzed by tSTAT1 and/or tSTAT2 Western blot. *n* = 3, one representative blot is presented. **(D)** Representative views of STAT1 ChIP-seq peaks detected in the ISRE-containing promoters of *Ifit1, Mx2, Oas2, Cxcl10*, and *Irf7* genes, in untreated or IFNα (8 h), IFNγ (8 h), LPS (4 h), IFNα (8 h) + LPS (4 h), IFNγ (8 h) + LPS (4 h)-stimulated VSMC. STAT1 peaks were mapped onto the mouse reference genome mm10 and visualized using the IGV genome browser. **(E)** VSMC were untreated or treated with IFNα (8 h) and IFNγ (8 h) and ChIP-PCR validation of tSTAT1 and pSTAT1 binding to ISRE motif present in the promoters at *Ifit1, Mx2, Oas2, Cxcl10*, and *Irf7* genes was performed. Mean ± s.e.m., *n* = 2. Primers are listed in Table S2. ChIP-PCR. VSMC were untreated or treated with IFNα (8 h) and IFNγ (8 h), chromatin was isolated and immunoprecipitated with **(F)** tSTAT2, pSTAT2, **(G)** IRF9 and **(H)** IRF1 antibodies, followed by ChIP-PCR analysis. Mean ± s.e.m., *n* = 2. Primers are listed in Table S2.

A striking observation after comparing IFNα + LPS and IFNγ + LPS commonly up-regulated genes was the high number of overlapping STAT1-binding ISRE-containing genes (Figure 2C). Close examination of the 45 ISRE-only and 59 ISRE-NFκB containing genes, up-regulated after stimulation with IFNγ + LPS (Figure 2C), identified the presence of an ISRE, but no GAS binding site, occupied by STAT1 in the regulatory regions of these genes. Moreover, STAT1 binding could already be observed after treatment of VSMC with IFNγ alone (data not shown), correlating with their transcriptional activity. Among these genes were classical ISRE-containing genes, from which we selected *Ifit1, Mx2, Oas2, Cxcl10*, and *Irf7* (Figure 3A) to further characterize the nature of this STAT1-dependent mechanism in several experiments. All 5 genes were highly responsive to IFNα and to a lesser extent to IFNγ, with *Ifit1, Mx2*, and *Cxcl10* being effected by SI after combined treatment with IFNα + LPS and IFNγ + LPS (Figure 3A). This correlated with the slight increase in STAT1 and STAT2 phosphorylation in response to both stimuli as compared to the individual ones (Figure 3B). *Ifit1, Mx2, Oas2*, and *Irf7* are examples of ISRE-only genes, which is in agreement with a single STAT1-binding peak (Figure 3D). In case of *Cxcl10* two STAT1-binding peaks were previously identified by Rauch et al., distal and proximal, corresponding to a known ISRE-GAS composite site and a single ISRE motif, respectively (9). Therefore, in this part of our study, the single ISRE site present in the proximal region of the *Cxcl10* promoter was chosen to further validate IFN-dependent STAT1 recruitment (Figure 3D). IGV genome browser views exhibited binding of STAT1 to ISRE-containing regions of all of these genes in VSMC, treated with IFNα, IFNγ, LPS, IFNα + LPS, and IFNγ + LPS (Figure 3D). STAT1 ChIP-seq results were further verified by quantitative ChIP-PCR, which demonstrated a significant enrichment of tSTAT1 recruitment to ISRE motifs present in the promoters of *Ifit1, Mx2, Oas2, Cxcl10*, and *Irf7* after stimulation with both IFNα and IFNγ (Figure 3E). This coincided with the binding of pSTAT1, which was significantly higher after IFNγ treatment than after IFNα treatment and reflected STAT1 phosphorylation levels under these conditions (Figure 3E). Similarly, we examined the potential binding of pSTAT2, tSTAT2, and IRF9 under these conditions (Figures 3F,G, respectively). It demonstrated increased recruitment of pSTAT2 to the ISRE-containing promoters of *Ifit1, Mx2, Oas2, Cxcl10*, and *Irf7* genes, after stimulation with IFNα and surprisingly with IFNγ (Figure 3F). Like pSTAT1, the level of pSTAT2 enrichment was in line with the STAT2 phosphorylation levels, which unexpectedly could also be detected after IFNγ and IFNγ + LPS treatment (Figure 3B). The binding of IRF9 showed a similar pattern as that of pSTAT2 and corresponded to IRF9 expression levels present in IFNα, IFNγ, and/or LPS treated cells (Figure 3B). The simultaneous recruitment of pSTAT1, pSTAT2, and IRF9 after IFNα and IFNγ treatment, clearly correlated with the involvement of ISGF3 in the transcriptional regulation of these ISRE-containing genes in response to both types of IFN. Indeed, co-IP of IRF9 with STAT1 and STAT2 in IFNα and IFNγ-treated VSMC corroborated this observation (Figure 3C). The expression pattern of these genes closely mirrored the binding pattern of pSTAT2 and IRF9, being higher after IFNα treatment in comparison to IFNγ treatment. Interestingly, the binding of STAT1 displayed an opposite pattern (higher after IFNγ treatment than after IFNα). This suggested the participation of STAT1 in an additional ISRE-binding complex in IFNγ-treated cells. Based on the high phosphorylation levels of STAT1 and the increased expression of IRF9 under these conditions, this complex could possibly consist of STAT1 homodimers together with IRF9 (39).

Since IRF1 expression levels increased in IFNα, IFNγ, and/or LPS treated VSMC (Figure 3B), we also tested the possible involvement of a STAT1-IRF1 containing complex. Interestingly, IRF1 was also recruited to these ISRE-containing genes after stimulation with IFNγ, but only weakly upon IFNα treatment (Figure 3H). The strongest IRF1 recruitment was noticed for *Ifit1* and *Mx2*, in comparison to *Oas2, Cxcl10*, and *Irf7* gene promoters. However, no interaction could be detected between STAT1 and IRF1 under these conditions (Figure 3C), pointing to a STAT1-independent role of IRF1 in the transcriptional regulation of a selective group of ISRE-containing genes.

Our results are in agreement with the existence of a more general mechanism in mouse primary VSMC, in which the IFNα response of ISRE-containing genes is mainly driven by ISGF3. In contrast, their IFNγ response is mediated by ISGF3 and potentially by STAT1/IRF9.

### Recruitment of STAT1 and p65 in Response to IFNα + LPS or IFNγ + LPS Is Restricted to GAS/NFκB or ISRE/NFκB Composite Sites

**Figure 4:**
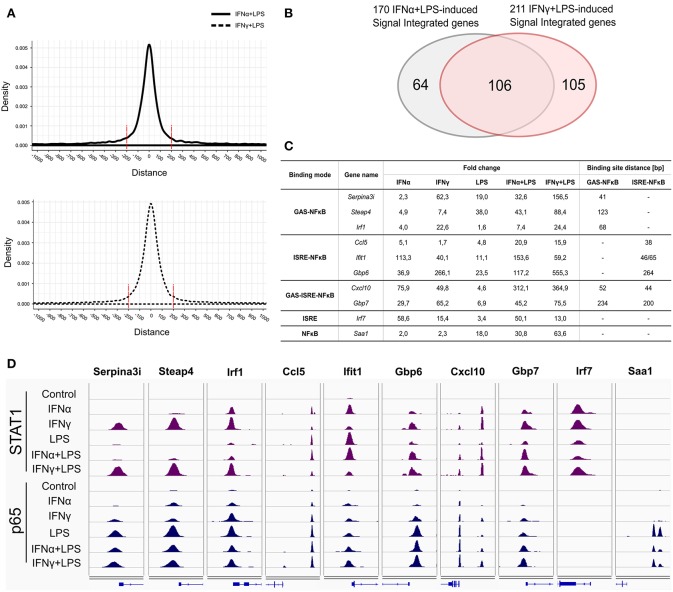
Representatives of STAT1 and p65 “single” and “co-binding” modes. **(A)** Peak distribution plots showing distances between summits of STAT1 and the closest p65 peak summits resulted from ChIP-seq in VSMC under IFNα (8 h) + LPS (4 h) and IFNγ (8 h) + LPS (4 h) treatment conditions. **(B)** Venn diagram showing the intersection of 170 IFNα (8 h) + LPS (4 h)- and 211 IFNγ (8 h) + LPS (4 h)-activated SI genes resulting from RNA-seq. **(C)** Table presents gene expression values (FC in comparison to control), resulting from RNA-seq experiment: VSMC treated with IFNα (8 h), IFNγ (8 h), LPS (4 h), IFNα (8 h) + LPS (4 h), IFNγ (8 h) + LPS (4 h) and GAS-NFκB and ISRE-NFκB binding sites distance (bp) for selected genes representing identified STAT1 and p65 ‘co-binding’ modes: GAS-NFκB: *Serpina3i, Steap4, Irf1*; ISRE-NFκB: *Ccl5, Ifit1, Gbp6*; GAS-ISRE-NFκB: *Cxcl10* and *Gbp7*; ISRE: *Irf7*; NFκB: *Saa1*. **(D)** Representative views of STAT1 and p65 ChIP-seq peaks (STAT1: violet peaks, p65: dark blue peaks) identified in the regulatory regions of *Serpina3i, Steap4, Irf1, Ccl5, Ifit1, Gbp6, Cxcl10, Gbp7, Irf7*, and *Saa1* genes, in untreated or IFNα (8 h), IFNγ (8 h), LPS (4 h), IFNα (8 h) + LPS (4 h), IFNγ (8 h) + LPS (4 h)-stimulated VSMC. STAT1- and p65-binding peaks were mapped onto the mouse reference genome mm10 and visualized using the IGV genome browser.

Subsequently, we concentrated on the overlap of STAT1-p65 “co-binding” modes between IFNα + LPS- and IFNγ + LPS-induced conditions. Interestingly, genome-wide these co-binding sites occurred at a similar distance of not more than ~200 bp (Figure 4A). First, we determined how many of the genes which were assigned either to GAS-NFκB, ISRE-NFκB, or GAS-ISRE-NFκB modes (Figure 2C) were affected by SI under these conditions. We identified 170 of such genes up-regulated by IFNα + LPS and 211 by IFNγ + LPS, of which 106 were in common (Figure 4B). From this list of genes, we selected several examples representing the three STAT1-p65 “co-binding” modes: *Serpina3i, Steap4, Irf1* (GAS-NFκB mode), *Ccl5, Ifit1, Gbp6* (ISRE-NFκB mode), *Cxcl10, Gbp7* (GAS-ISRE-NFκB mode). The RNA-seq FC values, representing gene expression changes upon treatment with IFNα, IFNγ, LPS, and IFNα + LPS, IFNγ + LPS, are presented in Figure 4C. Indeed, all of these genes were responsive to at least two single stimuli and affected by SI, reflected by increased gene expression after combined treatment with IFNα + LPS or IFNγ + LPS in comparison to the sum of the single treatments (Figure 4C).

STAT1 and p65 ChIP-seq IGV genome browser views of these pre-selected genes in response to IFNα + LPS and IFNγ + LPS, encompass the different STAT1-p65 “co-binding” modes (Figure 4D). IGV tracks reveal the binding pattern of STAT1 and p65 to the promoters of *Serpina3i, Steap4, Irf1* (GAS-NFκB sites), *Ccl5, Ifit1, Gbp6* (ISRE-NFκB sites), *Cxcl10, Gbp7* (GAS-ISRE-NFκB sites) genes, after stimulation with IFNα, IFNγ, LPS, and combined treatments with IFNα + LPS or IFNγ + LPS (Figure 4D). In case of *Cxcl10* two STAT1-p65 co-binding peaks could be observed (Figure 4D), distal and proximal, corresponding to a known GAS-ISRE-NFκB composite site and a combined ISRE-NFκB motif (9, 40). In this second part of our study, the GAS-ISRE-NFκB composite site present in the distal region of the *Cxcl10* promoter was chosen to further validate IFN-dependent STAT1 and p65 recruitment.

In conclusion, for the majority of these genes STAT1 and p65 binding peaks were closely aligned in the co-bound gene promoters, what further correlated with the close proximity of GAS and NFκB or ISRE and NFκB binding sites. This close binding sites distribution may be a pre-requisite for effective STAT1 and p65 collaboration.

### STAT1 Recruitment to GAS/NFκB or ISRE/NFκB Composite Sites Precedes p65 and Correlates With Elevated Transcription of IFNα + LPS and IFNγ + LPS Regulated Genes in VSMC

**Figure 5:**
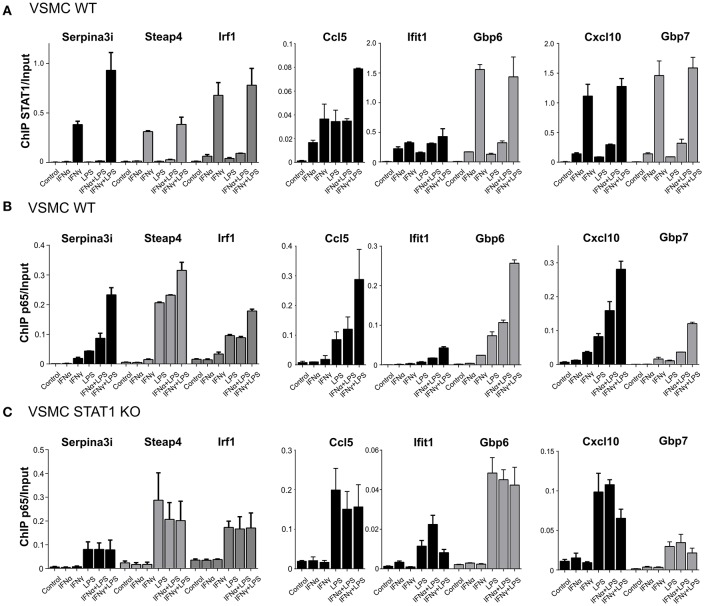
STAT1 modulates increased p65 recruitment to GAS/NFκB or ISRE/NFκB composite sites. **(A)** ChIP-PCR of STAT1 at *Serpina3i, Steap4, Irf1, Ccl5, Ifit1, Gbp6, Cxcl10*, and *Gbp7* gene promoters (primers are listed in Table S2) in VSMC WT treated with IFNα (8 h), IFNγ (8 h), LPS (4 h), IFNα (8 h) + LPS (4 h), IFNγ (8 h) + LPS (4 h). Mean ± s.e.m., *n* = 2. **(B)** ChIP-PCR of p65 at *Serpina3i, Steap4, Irf1, Ccl5, Ifit1, Gbp6, Cxcl10*, and *Gbp7* gene promoters in VSMC WT treated with IFNα (8 h), IFNγ (8 h), LPS (4 h), IFNα (8 h) + LPS (4 h), IFNγ (8 h) + LPS (4 h). Mean ± s.e.m., *n* = 2. **(C)** ChIP-PCR of p65 at *Serpina3i, Steap4, Irf1, Ccl5, Ifit1, Gbp6, Cxcl10*, and *Gbp7* gene promoters in VSMC STAT1 KO treated with IFNα (8 h), IFNγ (8 h), LPS (4 h), IFNα (8 h) + LPS (4 h), IFNγ (8 h) + LPS (4 h). Mean ± s.e.m., *n* = 2.

Validation experiments for STAT1 and p65 by quantitative ChIP-PCR, using freshly isolated material (Figures 5A,B) confirmed the binding pattern of both STAT1 and p65 as presented in Figure 4D. It also supported the following conclusions. First, for all genes, STAT1 and p65 binding peaks were closely aligned in the promoters. This correlated with the close proximity of GAS and NFκB or ISRE and NFκB binding sites (~200 bp; Figure 4C), which may be a pre-requisite for effective STAT1 and p65 collaboration. Moreover, Tables 1A,B confirm the presence of GAS, ISRE, and NFκB binding sites [indicated by a dot (•)] in the promoters of 30 of the highest commonly up-regulated genes by IFNγ + LPS and IFNα + LPS in VSMC. This additionally emphasizes the observation that the availability of multiple binding sites for these transcription factors within the gene promoters may play a role in coordination of immediate and robust gene transcriptional activation.

Second, although stimulation with both IFN-I and IFN-II resulted in elevated levels of total STAT1 protein, but not for total p65 (Figure 3B), for the majority of the genes the potency of STAT1 recruitment correlated with that of p65 binding. Interestingly, this was not restricted to IFNα + LPS and IFNγ + LPS treatments, but also clearly visible after single treatments with IFNα or IFNγ. In general, STAT1 and p65 binding after IFNγ and IFNγ + LPS was stronger than after IFNα and IFNα + LPS (Figures 5A,B). Third, increased p65 binding after single treatments with IFNα or IFNγ could only be detected at GAS/NFκB or ISRE/NFκB composite sites (Figure 4D), but not at genes with solitary NFκB binding sequences (exemplified by *Saa1*; Figure 4D).

Fourth, for all STAT1-p65 co-bound gene promoters except *Gbp6*, we observed a moderate increase in the recruitment of STAT1 after combined treatment with IFNγ + LPS in comparison to IFNγ alone. Likewise, combined treatment with IFNα + LPS resulted in slightly increased STAT1 binding in comparison to IFNα single treatment (Figure 5A). Notably, binding of STAT1 after IFNα treatment was significantly weaker in comparison to IFNγ-induced STAT1 recruitment, likewise to increased STAT1 recruitment after combined treatment. This observation correlated with FC expression values of examined STAT1-p65 “co-bound” genes (Figure 4C), which in general were more responsive to IFNγ + LPS than to IFNα + LPS. The same was true for p65, which recruitment, similar to STAT1 was increased after IFNα + LPS or IFNγ + LPS treatment in comparison to single LPS stimulation yet to a much higher extent (Figure 5B). In contrast, ChIP-PCR for p65 on chromatin isolated from untreated, IFNα, IFNγ, LPS, IFNα + LPS, IFNγ + LPS-treated STAT1 KO VSMC, for all genes resulted in abrogated recruitment of p65 after single treatments with IFNα or IFNγ (Figure 5C). Moreover, p65 binding remained unaltered after combined treatments with IFNα + LPS or IFNγ + LPS in comparison to LPS alone (Figure 5C).

Together, these observations could point to a STAT1-dependent role in the nearby recruitment of p65 upon single IFNα or IFNγ treatment, via closely located GAS and NFκB or ISRE and NFκB binding sites in the promoters of SI genes. More important, this STAT1-p65 co-binding was significantly increased upon subsequent LPS exposure and resulted in amplified transcriptional activity.

### IFNα + LPS and IFNγ + LPS Induced SI Correlates With Active Histone Marks and Increased PolII Recruitment in a STAT1-p65 Co-binding Dependent Manner

**Figure 6:**
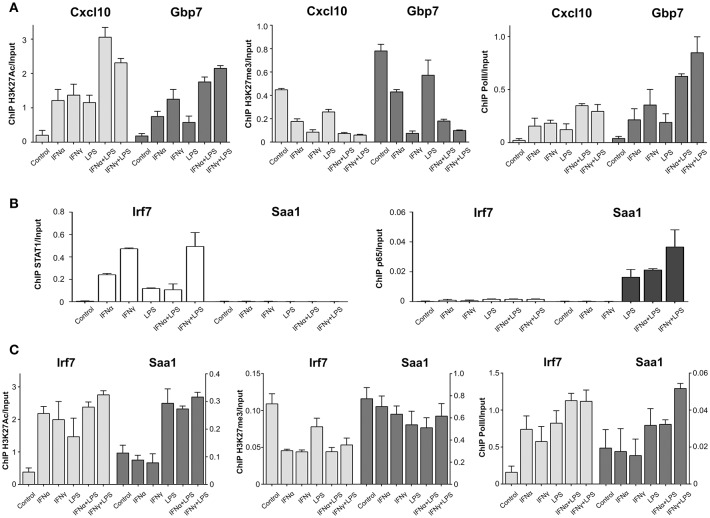
PolII and histone modification changes upon stimulation with IFNs and LPS at promoters of STAT1 and p65 “co-binding” and “single” modes representatives. **(A)** ChIP-PCR of H3K27Ac, H3K27me3, and PolII at *Cxcl10* and *Gbp7* gene promoters (primers are listed in Table S2) in VSMC treated with IFNα (8 h), IFNγ (8 h), LPS (4 h), IFNα (8 h) + LPS (4 h), IFNγ (8 h) + LPS (4 h). Mean ± s.e.m., *n* = 2. **(B)** ChIP-PCR of STAT1 and p65 at *Irf7* and *Saa1* gene promoters (primers are listed in Table S2) in VSMC treated with IFNα (8 h), IFNγ (8 h), LPS (4 h), IFNα (8 h) + LPS (4 h), IFNγ (8 h) + LPS (4 h). Mean ± s.e.m., *n* = 2. **(C)** ChIP-PCR of H3K27Ac, H3K27me3 and PolII at *Irf7* and *Saa1* gene promoters (primers are listed in Table S2) in VSMC treated with IFNα (8 h), IFNγ (8 h), LPS (4 h), IFNα (8 h) + LPS (4 h), IFNγ (8 h) + LPS (4 h). Mean ± s.e.m., *n* = 2.

To understand in more detail the epigenetic changes that coincide with STAT1 and p65 co-binding we investigated the establishment of active histone marks at the *Cxcl10* and *Gbp7* “co-binding” mode promoters. We observed increased enrichment of H3K27Ac at these promoters in response to IFNα, IFNγ, and LPS, which was further increased after IFNα + LPS and IFNγ + LPS treatment (Figure 6A). As expected, the binding pattern of the negative H3K27me3 mark was opposite to that of H3K27Ac under the different treatment conditions (Figure 6A). This could indicate that these genes harbor a permissive chromatin conformation, which is positively affected by IFNα + LPS and IFNγ + LPS stimulation. To address the mechanism of increased transcription after STAT1 and p65 co-binding we also analyzed the recruitment of PolII. As shown in Figure 6A, the association of PolII with the *Cxcl10* and *Gbp7* promoters mirrored that of the H3K27Ac mark and pointed to a STAT1-p65 co-binding dependent effect on histone acetylation and transcriptional activity upon IFNα + LPS and IFNγ + LPS treatment.

To further prove this, we analyzed the enrichment pattern of these two histone marks and PolII at the promoters of the “single” binding mode genes *Irf7* (STAT1 only) and *Saa1* (NFκB only) (Figures 4D, 6B). Indeed, H3K27Ac and PolII binding to the *Irf7* promoter showed an increase after IFNα, IFNγ, and LPS treatment. The opposite was true for H3K27me3 binding (Figure 6C), whereas the binding patterns for H3K27Ac, H3K27me3 and PolII did not significantly change after IFNα + LPS or IFNγ + LPS induction (Figure 6C). This was in line with STAT1 only binding, and no NFκB (Figures 4D, 6B), and the lack of SI on transcriptional activity (Figure 4C). For the *Saa1* promoter H3K27Ac association was not affected by IFNα or IFNγ treatment, but increased to a similar extent after LPS, IFNα + LPS, and IFNγ + LPS stimulation (Figure 6C). This coincided with NFκB only binding, and no STAT1 (Figures 4D, 6B). A similar, but only marginal LPS-mediated effect on abrogated H3K27me3 binding could be observed for the *Saa1* promoter (Figure 6C). PolII recruitment to the *Saa1* promoter exhibited a similar LPS-dependent pattern as that of H3K27Ac, although a slight increase was observed in response to IFNγ + LPS. This correlated with transcriptional activity of *Saa1*, which was substantially increased upon IFNα + LPS and IFNγ + LPS stimulation as compared to single stimuli (Figure 4C).

Our results suggest that STAT1 and p65 bind to DNA independently, yet in a sequential manner, directed by IFN-I or IFN-II treatment followed by LPS stimulation. As such, stimulation with IFNs results in robust STAT1 recruitment to ISRE and/or GAS motifs in gene promoters and potentially introduces chromatin modifications to increase NFκB binding to closely located sites and enhance transcription.

## Discussion

Excessive immune and inflammatory responses, communicated by immune, and vascular cells contribute to local inflammation and vascular dysfunction, followed by atherosclerotic plaque formation within the intima of the arterial wall. Priming-induced SI of IFN-II, and possibly IFN-I, with TLR4 is a common phenomenon in atheroma interacting immune cells that modulates important aspects of inflammation, with STAT1 and NFκB being central mediators. Thus, IFN pre-treatment (“priming”) followed by LPS stimulation leads to enhanced transcriptional responses as compared to the individual stimuli. To characterize the mechanism of priming-induced IFNα + LPS- and IFNγ + LPS-dependent SI in vascular cells as compared to immune cells, we performed a comprehensive genome-wide analysis of mouse VSMC, MΦ, and DC in response to IFNα, IFNγ, and/or LPS. Specifically, we aimed at providing mechanistic insight in the cooperative binding of STAT1 complexes with NFκB to ISRE/NFκB and/or GAS/NFκB binding sites in relation to transcription and how this is involved in the overlap of IFN-I/LPS and IFN-II/LPS activated SI in VSMC.

First, we compared the gene expression profiles of the different cell types exposed to the individual or combined stimuli, to identify the commonly up-regulated genes as a result of the interaction between IFNα and LPS or between IFNγ and LPS. Generally, in all three cell types combined treatment with IFNα + LPS or IFNγ + LPS resulted in a synergistic increase in gene expression as compared to single treatments, pointing to a common effect of SI mediated by the different IFNs (Figure 1). In agreement with the similar effect of SI mediated by the different IFNs and LPS, we observed >64% overlap between commonly up-regulated genes in response to IFNα + LPS and IFNγ + LPS. GO analysis revealed functional overlap of these genes connected to stress, immune and inflammatory response, response to cytokine, regulation of cell proliferation and migration, regulation of cell adhesion and chemotaxis, cell death and apoptotic process, response to lipid, and ROS metabolic process, all reflecting pro-inflammatory and pro-atherogenic biological functions (Figure 1). Together this may be a reflection of the partial overlap in activation of transcription factor complexes and regulation of target gene expression by IFN-I and IFN-II, which results in the execution of cell type-common biological responses. Subsequent promoter analysis of these genes indeed predicted the presence of either single GAS sites or rather combinations of potential GAS-ISRE, GAS-NFκB, or GAS-ISRE-NFκB binding motifs, with a similar binding site distribution between IFNα + LPS and IFNγ + LPS treatment conditions (Figure 1). In general, under these conditions ISRE motifs correspond to binding of STAT1 and STAT2 (in the form of ISGF3) and possibly different IRFs (IRF1, IRF7, IRF8, IRF9), GAS motifs to that of STAT1 binding and NFκB motifs to binding of p65 and p50. Previously we revealed, that the promoters of genes affected by the SI between IFNγ and LPS, like *Cxcl9, Cxcl10*, and *Nos2*, which are also present among the highest expressed common genes in the current study, contain STAT-NFκB and IRF-NFκB modules or combinations of separate ISRE, GAS or NFκB binding sites (20, 41). Together this suggested that a common SI mechanism is involved in the interaction between IFNα and LPS or IFNγ and LPS in VSMC, MΦ, and DC. Together this suggested that although VSMC, MΦ, and DC perform cell type specific functions in a healthy vessel, stimulation with pro-inflammatory stimuli results in activation of a common SI mechanism in the interaction between IFNα and LPS or IFNγ and LPS.

To understand this mechanism of SI in more detail, we characterized the genome-wide binding of STAT1 and NFκB (p65) to the IFNα + LPS- and IFNγ + LPS commonly up-regulated genes. Using ChIP-seq on chromatin from VSMC, STAT1 and p65 bound IFNα + LPS and IFNγ + LPS up-regulated genes were identified, containing GAS, ISRE, or NFκB binding motifs located in promoter regions, but also to up- and downstream genomic regions. Obviously, the interaction between IFNα + LPS and IFNγ + LPS increased the genome-wide number of STAT1 and p65 binding sites (as compared to individual treatments), correlating with the observed SI effect on transcription under these conditions (data not shown). The vast majority of STAT1 and p65 binding events localized outside gene promoters (Figure 2). This correlates with the general view of genome-wide occupancy of individual transcription factors, which regulate gene expression through integrated action of proximal and distal *cis*-regulatory elements [reviewed in (42)], the latter being functionally related with cell type-specific gene expression (43). This binding site distribution coincided with other studies. For example, in IFNγ stimulated HeLa S3 cells, Satoh et al. provided evidence for the presence of STAT1 binding GAS motifs in intronic regions (44), while others observed that ~50% of the total STAT1-occupied binding sites were intragenic and 25% intergenic (45). Similar observations have been reported for NFκB. As shown for LPS-treated THP1 cells, a significant proportion of genome-wide NFκB binding sites are located in proximal upstream promoter regions (26%), whereas an even greater proportion of p65 binding sites were found to be located within introns (38%) (46). On the other hand, in TNFα-treated HeLa cells, location analysis revealed that the p65-binding sites are mainly intragenic (46%) and only 7% are located in promoters, in agreement with previous studies (47). The function of the majority of distal STAT1 and p65 binding sites remains largely unknown. Nevertheless, it predicts the presence of a common regulatory mechanism of ISG transcriptional regulation.

Focusing on binding motifs in gene promoters, we could distinguish different STAT1 and p65 binding modes, including “single” (STAT1 binding to GAS and/or ISRE; p65 to NFκB) or “co-binding” (STAT1 binding to GAS and/or ISRE + p65 to NFκB) (Figure 2). Comparison of the different binding modes between IFNα + LPS and IFNγ + LPS induced conditions, identified a substantial overlap for NFκB-only (15.9%), GAS-only (13%), and ISRE-only (29.4%) containing genes from the single mode. Likewise, this overlap could be observed for GAS-ISRE (32.7%), GAS-NFκB (11.1%), ISRE-NFκB (21.6%), and GAS-ISRE-NFκB genes (29.6%) from the co-binding mode. A more detailed comparison of IFNα + LPS and IFNγ + LPS commonly up-regulated ISRE-containing genes identified STAT1 binding to these ISRE sites in response to IFNα and, unexpectedly to IFNγ (Figure 2). More important, this STAT1 DNA binding clearly corresponded to transcriptional activity in VSMC, as well as in MΦ and DC. Among these genes were many classical ISRE-containing genes, including *Ifit1, Mx2, Oas2, Irf7*, and *Cxcl10*, which were highly responsive to IFNα and to a lesser extent to IFNγ (Figure 3A). All five genes were highly responsive to IFNα and to a lesser extent to IFNγ, with *Ifit1, Mx2*, and *Cxcl10* being effected by SI after combined treatment with IFNα + LPS and IFNγ + LPS (Figure 3A). This correlated with the slight increase in STAT1 and STAT2 phosphorylation in response to both stimuli as compared to the individual ones (Figure 3B). The simultaneous recruitment of pSTAT1, pSTAT2, and IRF9 after IFNα and IFNγ treatment, clearly was in agreement with the involvement of ISGF3 in the transcriptional regulation of these ISRE-containing genes in response to both types of IFN. The expression pattern of these genes closely mirrored the binding pattern of pSTAT2 and IRF9 and reflected the phosphorylation level of STAT2 and expression of IRF9, being higher after IFNα treatment than after IFNγ treatment.

In support of a direct role for STAT2 in the IFNγ response, its tyrosine phosphorylation was reported in a study using IFNγ-treated wild-type mouse primary embryonic fibroblasts that caused the formation of ISGF3 (8). This was in agreement with observations made from the same group, in which mice lacking IRF9 are impaired not only in their type I IFN response, but also in their IFNγ-induced ISRE-dependent gene expression (48). Similar observations were made by others in MEFs, in which STAT2 phosphorylation was essential for the antiviral potency of IFNγ (49). Together, this revealed the existence of an ISGF3-dependent mechanism by which IFN-I and IFN-II can commonly elicit antiviral activities.

The opposite binding pattern of pSTAT1 (higher after IFNγ treatment than after IFNα; Figure 3), as compared to pSTAT2 and IRF9, suggested the participation of STAT1 in an additional ISRE-binding complex in IFNγ-treated cells. Based on the high phosphorylation levels of STAT1 and the increased expression of IRF9 under these conditions, we propose that this complex consists of STAT1 homodimers together with IRF9. The first evidence for STAT1- and IRF9-dependent and STAT2-independent transcriptional regulation of IFNγ-induced gene expression was reported for *Ifit2*, a classical ISRE-regulated gene (39) and CXCL10 (50). More recently a role for STAT1/IRF9 in the regulation of the latter gene was studied in the context of a murine colitis model. Molecular analysis in MΦ confirmed that STAT1/IRF9 complexes form in response to IFNγ and associate with ISRE sequences of enhancer regions 1 and 2 of the *Cxcl10* gene promoter (9). In the same study, the authors observed that the expression of IRF7 and DDX58, two other known ISRE-containing genes, depended on STAT1 and IRF9 as well as on STAT2 for their response to IFNγ pointing to a role of ISGF3 instead of STAT1/IRF9. As such they suggested that ISRE-containing promoters could potentially select STAT1/IRF9 complexes either with or without the STAT2 subunit for the cellular response to IFNγ. However, in the VSMC that we use in our study we cannot provide direct proof for a role of the STAT1/IRF9 complex in IFNγ-mediated responses in addition to ISGF3. Further experiments in VSMC from STAT2 and IRF9 KO mice will be needed to validate this assumption. Interestingly, IRF1 was also recruited to these ISRE-containing genes after stimulation with IFNγ, but only weakly upon IFNα treatment (Figure 3). Since no interaction could be detected between STAT1 and IRF1 under these conditions (Figure 3), a STAT1-independent role of IRF1 in the transcriptional regulation of a selective group of ISRE-containing genes can be proposed. This was in contrast to the direct interaction between unphosphorylated STAT1 and IRF1, which was detected in U3A cells overexpressing STAT1 tyrosine 701 mutant and proposed to mediate constitutive LMP2 gene expression (51).

**Figure 7:**
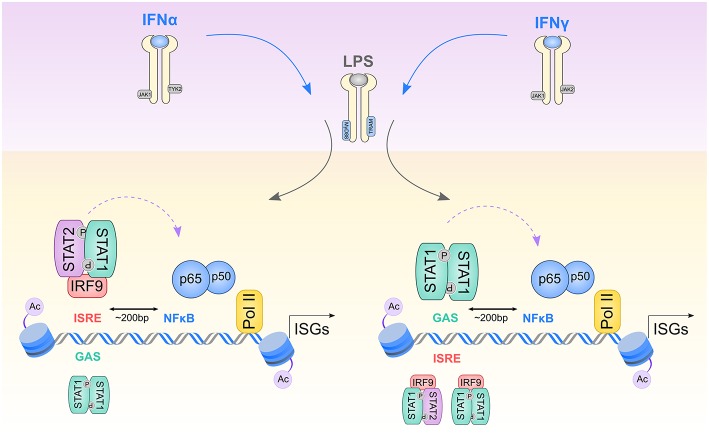
Model describing transcriptional regulation of Signal Integrated genes by STAT1-mediated preceding of p65 and PolII to acetylated GAS/NFκB or ISRE/NFκB composite sites. 1st wave of stimulation: After initial cell exposure to IFNα or IFNγ, receptors dimerize, and facilitate transphosphorylation of receptor-bound JAK1/TYK2 kinases for IFNα and JAK1/JAK2 kinases for IFNγ. Next STAT proteins are recruited, phosphorylated and dimerized, either in a form of ISGF3 complex (STAT1-STAT2 together with IRF9), GAF (STAT1 homodimers) or STAT1/IRF9 complex. Activated transcription factors supply a platform for 2nd wave of stimulation: LPS stimulates TLR4 receptor associated with adapter molecules MyD88 and TRAM and activates NFκB, as well as STAT1-containing transcriptional complexes. IFNα stimulation results in recruitment of ISGF3 to ISRE sites and GAF to GAS sites present in ISGs promoters. IFNγ initiates binding of GAF to GAS sites as well as ISGF3 and possibly STAT1/IRF9 to ISRE elements. Initial binding of STAT1-containing complexes followed by subsequent p65-p50 heterodimers binding (indicated by a violet curved arrow) to NFκB sites closely spaced to ISRE and GAS sites (~200 bp), together results in histone acetylation enrichment and PolII recruitment to ISG promoters. For a detailed explanation, see the text.

Our results are in agreement with the existence of a more general mechanism in mouse primary VSMC, in which ISGF3 and possibly STAT1/IRF9 regulate expression of IFNγ-responsive ISRE-containing genes. Together with STAT1 homodimers binding GAS, this provides an additional twist to the canonical IFNγ signaling pathway, which could explain some of the overlapping responses to IFNα and IFNy in these cells (Figure 7). Based on the overlapping expression patterns of these genes in VSMC, MΦ, and DC and the above described findings in MΦ, it is tempting to speculate that the IFNα response of ISRE-containing genes in all three cell types is mainly driven by ISGF3. In contrast, their IFNγ response is mediated by ISGF3 and potentially by STAT1/IRF9 (Figure 7). In the latter case a mechanism of competition could be envisioned or selective binding, depending on the ISRE sequence.

To further understand the mechanism of cooperative involvement of STAT1 with NFκB in SI mediated by the interaction of IFNα and LPS or IFNγ and LPS, we next concentrated on the overlap of STAT1-p65 co-binding modes. Therefore, we analyzed in more detail STAT1 and p65 binding patterns in 170 IFNα + LPS and 211 IFNγ + LPS up-regulated genes which were commonly affected by SI in the three cell types (Figure 4). Strikingly, for the majority of these genes STAT1 and p65 binding peaks were closely aligned in the co-bound gene promoters, what further correlated with the close proximity of GAS and NFκB (41–234 bp) or ISRE and NFκB (38–264 bp) binding sites (Figure 4). This close binding sites distribution may be a pre-requisite for effective STAT1 and p65 collaboration. A similar organization of closely located ISRE and NFκB sites, within ~50 bp proximity, was reported for IRF3 and NFκB co-occupancy to control Sendai virus-induced gene activation (52). Also in IFNγ-treated cells in genome-wide studies co-binding of STAT1 and IRF1 occurred at closely located GAS and ISRE sites (6, 53).

Stimuli-induced binding of two different transcription factors to closely spaced DNA motifs, could assume occurrence of direct protein-protein interactions, which if exceeding >20 bp would have to involve DNA looping (54). Indeed, STAT1 and NFκB have been shown to directly cooperate in several studies (55, 56). On the other hand, although combined action of STAT1 and NFκB was reported to be pivotal for *Cxcl9, IP-10, Becn1*, and NOS2 gene expression regulation, no direct protein-protein interaction of these transcription factors was observed (40, 57–59). Similarly, by performing co-IP experiments on protein extracts isolated from IFNα + LPS and IFNγ + LPS-treated VSMC, we were not able to detect direct interaction between STAT1 and p65 protein (data not shown). Further examination of the STAT1-p65 co-binding modes unraveled the involvement of a STAT1-dependent role in the nearby recruitment of p65 via closely located GAS-NFκB or ISRE-NFκB binding sites. IFNγ and to a lesser extent IFNα induced STAT1 binding to gene promoters containing either GAS-NFκB (*Serpina3i, Steap4, Irf1*), ISRE-NFκB (*Ccl5, Ifit1, Gbp6*), or GAS-ISRE-NFκB (*Cxcl10, Gbp7*) motifs. Much weaker STAT1 recruitment was also detected upon LPS stimulation, which correlated with the fact that transcriptional activation of SI genes under these conditions is primarily driven by IFNs (Tables 1A,B; Figures 4, 5). Interestingly, STAT1 recruitment to these different genes coincided with that of p65 binding, already upon IFNα or IFNγ treatment. This elevated p65 binding after single treatments with IFNα or IFNγ could not be detected at genes with solitary NFκB binding sequences (Figure 4). More important, subsequent LPS exposure resulted in increased STAT1-p65 co-binding, mainly driven by enhanced p65 recruitment, which correlated with histone acetylation, PolII recruitment and amplified target gene transcription in a STAT1-p65 co-bound dependent manner. In general, STAT1 and p65 binding after IFNγ and IFNγ + LPS was stronger than after IFNα and IFNα + LPS (Figure 5). The fact that we were not able to detect direct STAT1-p65 protein-protein interaction under studied treatment conditions in VSMC (data not shown), we postulate that STAT1 and p65 bind to DNA independently, yet in a sequential manner, directed by IFN-I or IFN-II treatment followed by LPS stimulation. As such, stimulation with IFNs results in robust STAT1 recruitment to ISRE and/or GAS motifs in gene promoters and potentially introduces chromatin modifications to increase NFκB binding to closely located sites.

Co-binding of STAT1 and NFκB has been studied in the context of bacterial infection. For example, sequential and cooperative contributions of NFκB preceding ISGF3, without direct protein-protein interaction, were shown to be involved in the transcriptional induction of the *Nos2* and *Il-6* genes in MΦ infected with the intracellular bacterial pathogen *Listeria monocytogenes*. In this context, NFκB acted as the major signal stimulated by TLR4 that introduced epigenetic marks to produce transcription friendly chromatin and enhanced subsequent recruitment of ISGF3, as the main signal from subsequent IFNβ production and action. This co-binding of NFκB followed by ISGF3, in combination with PolII, was a prerequisite for productive elongation of Nos2 and Il-6 mRNA (13, 60). Likewise, Wort et al. observed that combined stimulation of primary HPASM cells with TNFα and IFNγ correlated with both increased histone H4 acetylation at distinct NFκB sites and PolII recruitment to the PreproET-1 promoter region (61). Others showed that in IL-10 and LPS-treated phagocytes, STAT3 favored NFκB recruitment to the IL-1ra gene promoter due to its increased acetylation (62). A more comprehensive genome-wide co-binding study of IRF3 and NFκB revealed a mechanism of virus-induced transcriptional activation, in which IRF3 was able to organize promoter-specific recruitment of PolII and NFκB provided the ability to stimulate its efficient and processive elongation (52). On the other hand, Giorgetti et al. demonstrated p65 ability to additively recruit PolII to multiple κB sites containing gene promoters, resulting in elevated gene transcriptional activation (63). In case of IFNγ priming, a synergy mechanism was described, whereby IFNγ created a primed chromatin environment that sustained occupancy of STAT1, IRF1 and associated histone acetylation at pre-selected target genes. This greatly increased and prolonged recruitment of subsequent TLR4-induced transcription factors, including NFκB, and PolII to gene promoters and enhancers (64).

Based on these models our results are predictive of the following mechanism of STAT1-NFκB co-binding involved in the SI of IFN-I and IFN-II with TLR4 in VSMC, MΦ, and DC (Figure 7). In the first step, IFN-I activated STAT1 is recruited to closely located ISRE-NFκB or GAS-NFκB binding sites in the form of ISGF3 or GAF, respectively. Likewise, IFNγ stimulates the binding of the STAT1-complexes ISGF3 (and possibly STAT1/IRF9) and GAF to these respective sites. This first wave of STAT1 binding introduces chromatin modifications and initiates subsequent p65-p50 recruitment to adjacent (~200 bp) NFκB sites in response to IFNγ and to a lesser extent after IFNα treatment, which correlates with STAT1-binding potency and levels of transcription. The second step, which is mediated by subsequent LPS stimulation, increases STAT1-p65 co-binding to these different composite sites and is mainly driven by enhanced p65-p50 dimer formation and recruitment. This coincides with histone acetylation, PolII recruitment and amplified transcription of IFNα + LPS and IFNγ + LPS up-regulated genes, which in general is stronger after IFNγ + LPS than after IFNα + LPS (Figure 7). In case of genes harboring GAS-ISRE-NFκB composite sites, similar but more complex mechanisms of canonical and non-canonical STAT1 complexes in response to IFN-I or IFN-II combined with LPS-activated NFκB are probably involved.

Thus, transcription factor complexes activated by IFN-I or IFN-II together with LPS, including GAF, ISGF3, STAT1/IRF9, and p65-p50 heterodimers provide a platform for robust transcriptional activation of pro-inflammatory genes. Moreover, our model offer for the first time an explanation for the comparable effects of IFNα or IFNγ priming on TLR4-induced activation in vascular and immune cells, which correlates with the important roles of both IFN types in vascular inflammation and atherosclerosis progression. However, we realize that this is just a predictive model and we cannot rule out the involvement of other STAT1-containing transcription factor complexes or IRFs. Moreover, further investigation will be required to obtain insight in the mechanism of STAT1-dependent NFκB recruitment and subsequent transcriptional regulation under SI, involved in gene-specific scenarios.

## Author Contributions

AP-B performed ChIP-seq, ChIP-PCR, Western blot and Co-IP experiments, RNA-seq/ChIP-seq downstream *in silico* analysis and was involved in concept development, writing, figures and tables preparation, and editing manuscript. LS and AC performed *in silico* ChIP-seq analysis. H-CC isolated RNA from DC for further RNA-seq experiment and performed RT-PCR validation. C-KL and LN were involved in concept development related to DC and MΦ experimentation. JW critically assessed the manuscript. HB developed the research study concept and was involved in data interpretation, writing and editing the manuscript, and coordinated input from all co-authors.

### Conflict of Interest Statement

The authors declare that the research was conducted in the absence of any commercial or financial relationships that could be construed as a potential conflict of interest.

## Abbreviations

ChIP: Chromatin Immunoprecipitation
Co-IP: Co-immunoprecipitation
DC: Dendritic cells
EC: Endothelial cells
GAF: γ-activated factor
GO: Gene ontology
IFN: Interferon
IRF: Interferon Regulatory Factor
ISG: IFN-stimulated gene
ISGF3: Interferon-stimulated gene factor 3
ISRE: Interferon-stimulated response element
JAK: Janus kinase
LPS: Lipopolysaccharide
MΦ: Macrophages
NFκB: Nuclear Factor-κB
PolII: RNA polymerase II
ROS: Reactive oxygen species
SI: Signal Integration
STAT: Signal Transducer and Activator of Transcription
TNF: Tumor necrosis factor
TLR: Toll-like receptor
VSMC: Vascular Smooth Muscle Cells.
