# Supplementary material for: Signal Integration of IFN-I and IFN-II With TLR4 Involves Sequential Recruitment of STAT1-Complexes and NFκB to Enhance Pro-inflammatory Transcription
Source: Front Immunol. 2019 Jun 4;10:1253. doi: 10.3389/fimmu.2019.01253 (PMC6558219; doi:10.3389/fimmu.2019.01253)
Supplement: Supplementary file 1 [file Data_Sheet_1.PDF]

Supplementary Material

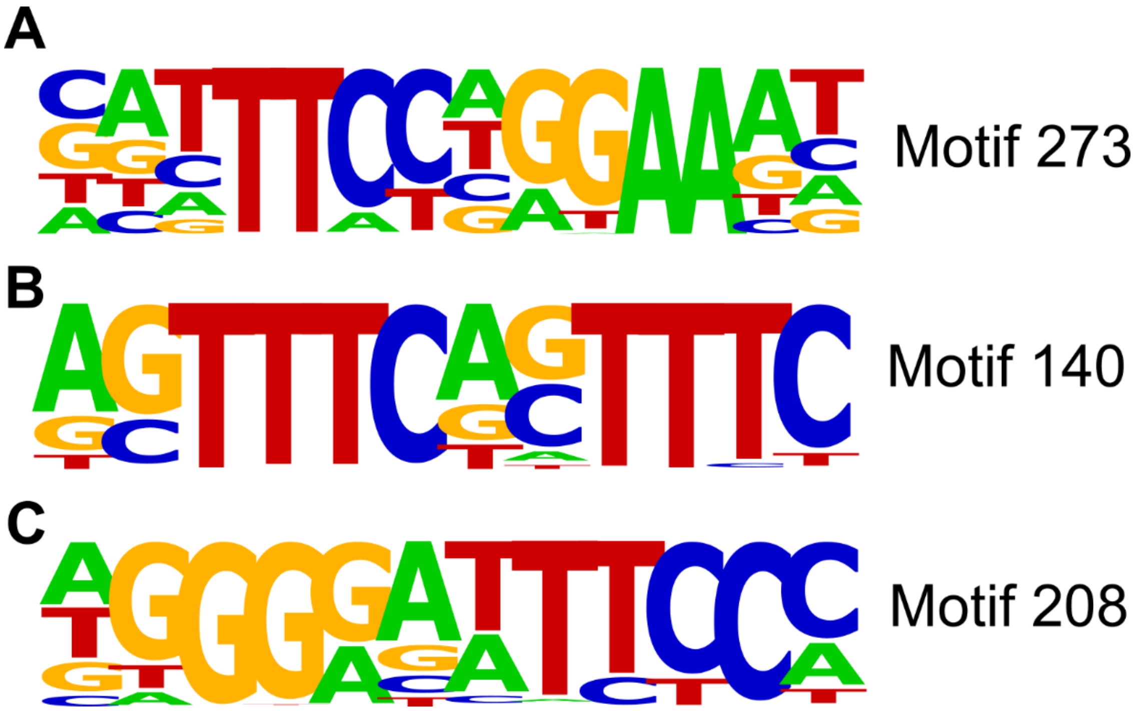

Figure S1. Logo representations of HOMER Motif Database motifs used for re-mapping analysis in STAT1 and p65 ChIP-seq experiments.
(A) Sequence logo (motif 273) representing GAS motif. **(B)** Sequence logo (motif 140) representing ISRE motif. **(C)** Sequence logo (motif 208) representing NFκB-p65 motif.

Table S1. Lists of up-regulated genes (FC>2) by IFNα, IFNγ, LPS, IFNα+LPS and IFNγ+LPS in VSMC, MΦ and DC. These 15 lists, resulting from RNA-seq experiment, were further compared by Venn diagram analysis.
(provided in separate excel file)

Table S2. List of primer sequences used in ChIP-PCR experiments.

Gene name	Binding site	Primer sequence	
		Forward	Reverse
Ccl5	ISRE/NFκB	CTGCAGCCAAAGAAACCGAAA	ACACAGTCATGGGGAAACCC
Cxcl10_1	ISRE/NFκB	AGTTTCCCTCCCTGAGTCCT	ACAAGCAATGCCCTCGGTTT
Cxcl10_2	GAS/ISRE/NFκB	TAACTGCGAGTGAGCACAGA	TAGGACATTGGACAGAGCGG
Gbp6_1	ISRE	AGAGAGAAGGAGTTAAAAGAAATCACA	CATGGCCTATTTTATTTTACTTTCAGT
Gbp6_2	NFκB	AAGTGTGCTTCTCTCAGGGG	TGGTTCCTGCTTTAGAAAACACAT
Gbp7	GAS/ISRE/NFκB	AGGAGCTGGCACATTTCTGT	TTGCCTGCACTTAGTTGTGG
Ifit1	ISRE/NFκB	GATTTCAGTGGAGAATGCAGTAGG	GTGTGCTCTTTTCAGTCAGCAGT
Irf1	GAS/NFκB	CTTCGCCGCTTAGCTCTACA	TGAAAGCACGTCCTACCTCG
Irf7	ISRE	TGGTAGGCATGGAGACAGTG	AAACGAAACTGCATCTCAGGA
Mx2	ISRE	TTCCCCAAGAACCAGAGAAA	CCTCTCTCCCTGTTGCCTTT
Oas2	ISRE	AACACAGCCAAGCCTAGGAA	GCTAGCTGGAAGCAAACACAC
Saa1	NFκB	TGTGCATAGTGTCTGGGGAAA	ACAATTAGTGGAAGTGGCCCG
Serpina3i	GAS/NFκB	CTGGGGAAATGTGGTCTGTGTT	TCTGGGCTCTGATGGGAAAAG
Steap4	GAS/NFκB	ATCTCTCTGCCCCTCTAGGC	CCCGCTTGATTTCGCAAGAG
